# Aerial Cooperative Jamming for Cellular-Enabled UAV Secure Communication Network: Joint Trajectory and Power Control Design

**DOI:** 10.3390/s19204440

**Published:** 2019-10-14

**Authors:** Hanming Sun, Bin Duo, Zhengqiang Wang, Xiaochen Lin, Changchun Gao

**Affiliations:** 1 The Glorious Sun School of Business and Management, Donghua University, Shanghai 200051, China; 1149151@mail.dhu.end.cn (H.S.); gcc369@dhu.edu.cn (C.G.); 2 College of Information Science & Technology, Chengdu University of Technology, Chengdu 610059, China; 3 School of Communication and Information Engineering, Chongqing University of Posts and Telecommunications, Chongqing 400065, China; wangzq@cqupt.edu.cn; 4 School of Electronic Information Engineering, Shanghai Dianji University, Shanghai 201306, China; linxc@sdju.edu.cn

**Keywords:** UAV secure communication, secrecy rate maximization, jamming, trajectory design, power control

## Abstract

To improve the secrecy performance of cellular-enabled unmanned aerial vehicle (UAV) communication networks, this paper proposes an aerial cooperative jamming scheme and studies its optimal design to achieve the maximum average secrecy rate. Specifically, a base station (BS) transmits confidential messages to a UAV and meanwhile another UAV performs the role of an aerial jammer by cooperatively sending jamming signals to oppose multiple suspicious eavesdroppers on the ground. As the UAVs have the advantage of the controllable mobility, the objective is to maximize the worst-case average secrecy rate by the joint optimization of the two UAVs’ trajectories and the BS’s/UAV jammer’s transmit/jamming power over a given mission period. The objective function of the formulated problem is highly non-linear regarding the optimization variables and the problem has non-convex constraints, which is, in general, difficult to achieve a globally optimal solution. Thus, we divide the original problem into four subproblems and then solve them by applying the successive convex approximation (SCA) and block coordinate descent (BCD) methods. Numerical results demonstrate that the significantly better secrecy performance can be obtained by using the proposed algorithm in comparison with benchmark schemes.

## 1. Introduction

Due to the many advantages of controllable mobility, such as on-demand fast deployment, wide coverage, low cost, and line-of-sight (LoS) transmission that offers good channel capacity, unmanned aerial vehicles (UAVs) have been extensively utilized in different scenarios, e.g., surveillance and monitoring [1,2,3], search and rescue [4,5], cargo transportation [6], data collection [7] and mobile relays [8].

Recently, UAVs have attracted increasing attention in wireless communications, and are anticipated to playing an important role in the next-generation wireless networks [9,10]. Generally, there are two promising solutions to UAV communication applications: cellular-enabled UAV communication (CEUC) and UAV-assisted terrestrial communication (UATC) networks [11]. In UATC, the UAVs are flexibly deployed as aerial base stations (BSs) or mobile relays to assist in providing reliable communication services for terrestrial networks [12,13,14]. By contrast, the UAVs are integrated into the wireless network scenarios as aerial users served by ground BSs in the CEUC system [15]. Owing much to the almost ubiquitous accessibility of the existing LTE (Long Term Evolution) and the forthcoming (beyond) fifth-generation ((B)5G) cellular networks, reliable communications can be supported between UAVs and their corresponding BSs [16,17]. The CEUC is anticipated to have a number of appealing advantages over the existing ground-to-UAV communications, including the ease of monitoring and management, ubiquitous accessibility, robust navigation and enhanced performance, etc. [11]. Despite its merits, the UAV communication based on the future (B)5G cellular networks is more susceptible to suspicious eavesdropping on the ground, which leads to a severe security challenge that is urged to be solved.

Currently, the UAV trajectory design combined with physical-layer security techniques, as a promising solution, has drawn significant attention to safeguard the UAV communication. Specifically, UAV secure communications are studied in [18,19], where the average secrecy rate is significantly improved via optimizing the trajectory of the UAV jointly with the power control for a finite mission duration. As cooperative jamming is one of the important physical-layer techniques that can enhance the secrecy performance, reference [20] proposed to employ a UAV as a friendly mobile jammer, to ensure the secrecy of the ground wiretap channel. In [21], a novel full-duplex operation was applied to the rotary-wing UAV to further improve the energy efficiency (EE) of UAV secrecy communications, and the EE was maximized by the joint optimization of the source transmit/UAV’s jamming power and UAV trajectory. A four-node mobile relay and eavesdropper system is proposed in [22], where the UAV was employed as a mobile relay to assist in terrestrial communications. To cope with the non-convex secrecy rate maximization problem, an alternating optimization algorithm is designed by optimizing the power control and UAV trajectory alternatively. The authors in [23,24] proposed a dual-UAV UATC network to enhance the communication quality and improve the secrecy performance, where the downlink transmission from the UAVs is established by adaptively adjusting the UAVs’ trajectories and transmit powers. Note that most of the above studies only focus on the security issues in UATC systems. However, how to design efficient anti-eavesdropping methods, to protect legitimate BS-to-UAV transmission in the CEUC networks, has not been investigated, and thus remains a challenging problem to address.

In light of the above, we propose an anti-eavesdropping scheme by employing an aerial UAV jammer in the CEUC network, where one UAV flies to receive confidential messages from a BS while the mobile UAV jammer confuses multiple suspicious eavesdroppers on the ground by sending jamming signals. Specifically, we take into account the joint optimization of both the UAVs’ trajectories and the BS’s/UAV jammer’s power allocation, in order to maximize the average worst-case secrecy rate of the UAV receiver for a given finite period. In the proposed scheme, the UAVs are subject to the practical mobility as well as both the average and peak power constraints. In contrast with the above-mentioned existing works, the UAVs’ trajectory design in our proposed CEUC network is particularly important, as the interference from other UAVs cannot be practically cancelled, which causes different objective function and constraints. Therefore, well-designed trajectories of the UAVs can not only avoid severe interference between UAVs, but also provide effective jamming signals to the eavesdroppers, which is expected to notably enhance the secrecy performance. As the formulated optimization problem is non-convex with the objective function as well as its constraints, it is very hard to obtain a globally optimal solution (Since the difficulty of the original problem is NP-hard, it is generally impossible to obtain the globally optimal solution by using the present optimization techniques.) To tackle this challenging problem, we first transform it into a lower bound expression with more tractability. Then, an efficient algorithm is designed by applying the block coordinate descent (BCD) method [25,26]. To be specific, we partition the total optimization variables into four blocks for the two UAVs’ trajectories, BS’s transmit power, and UAV jammer’s jamming power control, respectively. Then, each block is alternatively optimized in each iteration with other blocks being fixed. Although we fix the other three blocks, the corresponding optimization problem remains intractable because of its non-convex. To obtain a high-quality approximately optimal solution, we thus introduce a series of slack variables and apply successive convex approximation (SCA) technique [27,28]. The proposed algorithm has the applicable complexity and guarantees to converge to a locally optimal solution to this problem. To best of knowledge, this is the first work that exploits the anti-eavesdropping UAV trajectory design to solve physical-layer security issues of the CEUC system. The numerical results illustrate that the designed algorithm achieves significantly better secrecy performance than all benchmarks without trajectory or power control design, especially the scheme without the UAV jammer, as in [18].

The rest of this paper is organized as follows. Section 2 gives the system model and problem formulation. In Section 3, a joint optimization algorithm is proposed and its complexity and convergence performance are also analyzed. The simulations are presented in Section 4 to verify the effectiveness of the proposed algorithm. Finally, Section 5 concludes the paper.

## 2. System Model and Problem Formulation

### 2.1. System Model

**Figure 1:**
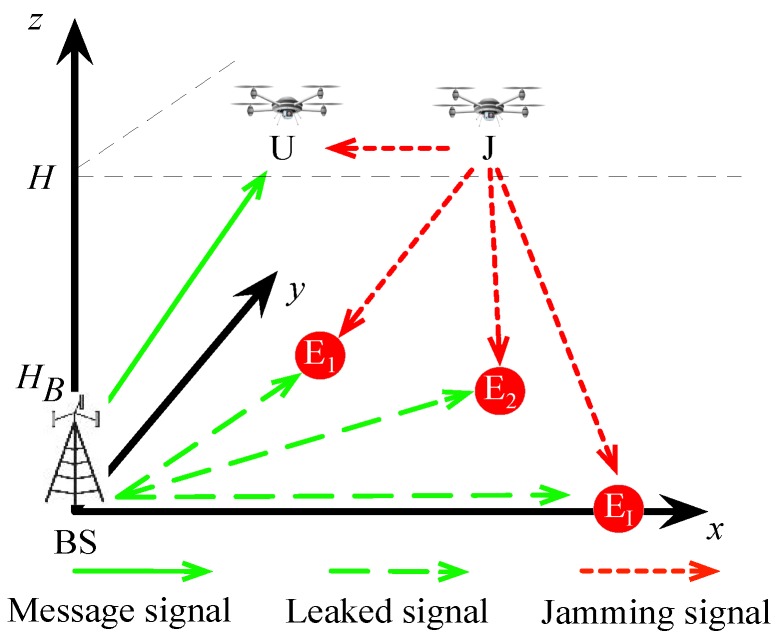
Cellular-enabled UAV secure communication network with aerial cooperative jamming.

Consider a CEUC network, as shown in Figure 1, where a ground BS transmits confidential messages to a mobile UAV receiver (denoted by U) within a given UAV flight period *T*, while *I* malicious eavesdroppers on the ground, denoted by $E_{i}$ for i∈I≜{1,⋯,I}, intercept the messages from the valid UAV communication. To safeguard the legitimate transmission, the potential eavesdroppers are kept under surveillance by an aerial UAV jammer (denoted by J). The aim of the UAV J is to cooperatively send jamming signals to the eavesdroppers to resist their wiretapping. Notice that if there is no friendly UAV J and only one eavesdropper is considered, the proposed scenario reduces to the goround-to-UAV transmission in [18].

Based on the three-dimensional Cartesian coordinate system, we denote $w_{B}={\left[x_{B},y_{B}\right]}^{T}$ and $w_{E_{i}}={\left[x_{E_{i}},y_{E_{i}}\right]}^{T}$ as the horizontal coordinates of the BS and $E_{i}$, respectively, which are assumed to be fixed and known beforehand to the UAVs. The assumption that $w_{E_{i}}$ is known in the network is proper when $E_{i}$ is an active ground node but untrusted by the UAV [29]. Therefore, $E_{i}$ can be detected by the synthetic aperture radar or optical camera mounted on the UAV [18]. The initial and final locations of the UAVs are assumed to be pre-specified, which are denoted by $q_{k,0}={\left[x_{k,0},y_{k,0}\right]}^{T}$ and $q_{k,F}={\left[x_{k,F},y_{k,F}\right]}^{T}$ for k∈{U,J}, respectively. To make it more manageable, the period *T* is partitioned into *N* equal-length time slots, i.e., $T=\delta_{t}N$, where $\delta_{t}$ is the length of one time slot. As such, the UAV trajectory in time slot n∈N can be represented approximately by $q_{k}\left[n\right]≜{\left[x_{k}\left[n\right],y_{k}\left[n\right]\right]}^{T}$ for k∈{U,J}, with a fixed altitude *H*. Let $\Omega =V_{\max}\delta_{t}$ be the maximum horizontal distance that the UAV can travel in a single time slot, where $V_{\max}$ is the maximum speed of the UAV. Practically, the UAVs should satisfy the following mobility constraints,

(1) $$\left|\right|q_{k}\left[n+1\right]-q_{k}\left[n\right]{\left|\right|}^{2}\leq \Omega^{2},n=0,\cdots ,N-1,$$

(2) $$q_{k}\left[0\right]=q_{k,0},q_{k}\left[N\right]=q_{k,F},$$

(3) $$\left|\right|q_{U}\left[n\right]-q_{J}\left[n\right]{\left|\right|}^{2}\geq d_{\min}^{2},n=0,\cdots ,N,$$

where $d_{\min}$ is the minimum tolerable distance between the two UAVs that ensures the avoidance of a collision.

We assume that the ground-to-UAV and UAV-to-UAV transmissions are mainly governed by LoS channels [18,20,23,24]. Thus, the corresponding channel power gains in time slot *n* follow the free-space path loss model. (For the purpose of exposition, it is reasonable to assume that the ground-to-UAV follows the free-space LoS channel model when the UAV is deployed in the rural area with sufficiently high altitude. In this case, the probability of Non-LoS state is negligible compared to the dominant LoS state [30]. However, the proposed design is readily extendable to more general channel models in urban areas with Non-LoS effects, e.g., [30].), which are, respectively, given as below,

(4) $$h_{\mathrm{BU}}\left[n\right]=\rho_{0}d_{\mathrm{BU}}^{-2}\left[n\right]=\frac{\rho_{0}}{{\left(H-H_{B}\right)}^{2}+\left|\right|q_{U}\left[n\right]-w_{B}{\left|\right|}^{2}},$$

(5) $$h_{{\mathrm{JE}}_{i}}\left[n\right]=\rho_{0}d_{{\mathrm{JE}}_{i}}^{-2}\left[n\right]=\frac{\rho_{0}}{H^{2}+\left|\right|q_{J}\left[n\right]-w_{E_{i}}{\left|\right|}^{2}},$$

(6) $$h_{\mathrm{JU}}\left[n\right]=\rho_{0}d_{\mathrm{JU}}^{-2}\left[n\right]=\frac{\rho_{0}}{\left|\right|q_{U}\left[n\right]-q_{J}\left[n\right]{\left|\right|}^{2}},$$

where $d_{\mathrm{BU}}\left[n\right]$, $d_{{\mathrm{JE}}_{i}}\left[n\right]$ and $d_{\mathrm{JU}}\left[n\right]$ are the distances from the BS to the UAV U, from the UAV J to the eavesdropper $E_{i}$, and between the two UAVs in time slot *n*, respectively, $\rho_{0}$ is the channel power gain at the reference distance $d_{0}=1$ m and $H_{B}$ is the altitude of the BS. The ground-to-ground transmission is assumed to follow the Rayleigh fading channel. As such, the channel power gain is denoted by

(7) $$h_{{\mathrm{BE}}_{i}}=\rho_{0}\zeta_{i}d_{BE_{i}}^{-\kappa }=\frac{\rho_{0}\zeta_{i}}{\left|\right|w_{B}-w_{E_{i}}\left|\right|{}^{\kappa }},$$

where $d_{BE_{i}}$ is the distance between the BS and the eavesdropper $E_{i}$, $\zeta_{i}$ is an exponentially distributed random variable with unit mean representing small-scale Rayleigh fading and κ≥2 is the distance-dependent path loss exponent.

Denote by P[n] and Q[n] the BS’s transmit power and the UAV J’s jamming power in time slot *n*, respectively. In practice, they should satisfy the respective average power constraint $\bar{P}$ or $\bar{Q}$, and peak power constraint $\hat{P}$ or $\hat{Q}$, i.e.,

(8) $$\frac{1}{N}\sum_{n=1}^{N}P\left[n\right]\leq \bar{P},0\leq P\left[n\right]\leq \hat{P},$$

(9) $$\frac{1}{N}\sum_{n=1}^{N}Q\left[n\right]\leq \bar{Q},0\leq Q\left[n\right]\leq \hat{Q},$$

where $\bar{P}\leq \hat{P}$ and $\bar{Q}\leq \hat{Q}$. Then, the achievable rate in bits/second/Hertz (bps/Hz) of the UAV U in time slot *n* is given by

(10) $$\begin{array}{cc} R_{U}\left[n\right] & =\log_{2}\left(1+\frac{P\left[n\right]h_{\mathrm{BU}}\left[n\right]}{Q\left[n\right]h_{\mathrm{JU}}\left[n\right]+\sigma^{2}}\right), \end{array}$$

where $Q\left[n\right]h_{\mathrm{JU}}\left[n\right]$ is the jamming interference from the UAV J, and $\sigma^{2}$ is the additive white Gaussian noise power at the receivers. Similarly, the achievable rate of the eavesdropper $E_{i}$ in time slot *n* can be expressed as

(11) $$\begin{array}{cc} R_{E_{i}}\left[n\right] & =E_{\zeta_{i}}\left[\log_{2}\left(1+\frac{P\left[n\right]h_{{\mathrm{BE}}_{i}}}{Q\left[n\right]h_{{\mathrm{JE}}_{i}}\left[n\right]+\sigma^{2}}\right)\right]\\ \; & \leq \log_{2}\left(1+\frac{P\left[n\right]\rho_{0}\left|\right|w_{B}-w_{E_{i}}\left|\right|{}^{-\kappa }}{Q\left[n\right]h_{{\mathrm{JE}}_{i}}\left[n\right]+\sigma^{2}}\right)\\ \; & ≜\hat{R}{}_{E_{i}}\left[n\right], \end{array}$$

where $E_{\zeta_{i}}\left[\cdot \right]$ is the expectation operator with respect to (w.r.t.) $\zeta_{i}$. Note that $R_{E_{i}}\left[n\right]$ is replaced by $\hat{R}{}_{E_{i}}\left[n\right]$ based on Jensen’ inequality and the concavity of $R_{E_{i}}\left[n\right]$ w.r.t. $\zeta_{i}$, and $\hat{R}{}_{E_{i}}\left[n\right]$ is the largest rate that $E_{i}$ can achieve. Therefore, in accordance with the theoretical results in [31], the worst-case secrecy rate for each time slot can be lower bounded by

(12) $$R_{\mathrm{wcs}}\left[n\right]=\max\left(R_{U}\left[n\right]-\max_{i\in I}\hat{R}{}_{E_{i}}\left[n\right],0\right).$$

Note that by adaptively setting P[n]=0, the optimal solution to (12) is at least to be zero for any time slot *n*, without violating the power constraint (8). Therefore, the maximum operation can be dropped in the following optimization problems.

### 2.2. Problem Formulation

In this paper, we aim to maximize the average worst-case achievable secrecy rate from the BS to the UAV U over *N* time slots, by jointly optimizing the BS’s transmit power P≜{P[n],n∈N}, the jamming power Q≜{Q[n],n∈N} of the UAV J, and the UAV trajectory $q_{k}=\left\{q_{k}\left[n\right],n\in N\right\}$ for k∈{U,J}. Thus, this optimization problem can be formulated as

(13) $$\max_{P,Q,q_{U},q_{J}}\frac{1}{N}\sum_{n=1}^{N}\left(R_{U}\left[n\right]-R_{E}\left[n\right]\right)$$

s.t.(1)–(3),(8)–(9).

where we let $R_{E}\left[n\right]=\max_{i\in I}\hat{R}{}_{E_{i}}\left[n\right]$, and thus $R_{E}\left[n\right]$ corresponds to the maximum achievable rate among multiple eavesdroppers in time slot *n.* Optimally solving problem (13) is difficult, in general, due to the following two main reasons: (1) the objective function is not concave w.r.t the corresponding optimization variables even with fixed variables of other blocks and (2) the constraint in (3) is non-convex w.r.t. the UAVs’ trajectory variables.

## 3. Joint Trajectory and Power Control Algorithm

In this section, an efficient algorithm is proposed to obtain the sub-optimal solution to problem (13). Specifically, we cope with problem (13) by solving four subproblems iteratively, i.e., the alternative optimization of the transmit power P, jamming power Q, UAV U’s trajectory $q_{U}$, and UAV J’s trajectory $q_{J}$, by fixing the other three optimization variables. Furthermore, the overall algorithm is presented, and its complexity and convergence are analyzed rigorously.

### 3.1. Transmit Power Optimization

For simplicity, let $a_{n}=\frac{\gamma_{0}}{d_{\mathrm{BU}}^{2}\left[n\right]\left(1+Q\left[n\right]\gamma_{0}/\rho_{0}d_{\mathrm{JU}}^{2}\left[n\right]\right)}$, and $b_{n}=\frac{\gamma_{0}\left|\right|w_{B}-w_{E}\left|\right|{}^{-\kappa }}{1+Q\left[n\right]\gamma_{0}/d_{\mathrm{JE}}^{2}\left[n\right]}$, where $\gamma_{0}=\rho_{0}/\sigma^{2}$ is the reference signal-to-noise ratio (SNR), and $w_{E}$ is denoted as the horizontal location of the eavesdropper that achieves the largest rate and $d_{\mathrm{JE}}^{2}\left[n\right]$ is the distance from the UAV J to the eavesdropper $w_{E}$. Thus, with given Q, $q_{U}$, and $q_{J}$, problem (13) can be simplified as

(14) $$\max_{P}\sum_{n=1}^{N}\left[\log_{2}\left(1+a_{n}P\left[n\right]\right)-\log_{2}\left(1+b_{n}P\left[n\right]\right)\right]$$

s.t.(8).

Based on the result in [18], the close-form solution to this problem is given by: $P^{*}\left[n\right]=\min\left({\left[\Lambda_{n}\right]}^{+},\hat{P}\right)$ if $a_{n}>b_{n}$; otherwise $P^{*}\left[n\right]=0$, where $\Lambda_{n}={\left({\left(1/2b_{n}-1/2a_{n}\right)}^{2}+\left(1/b_{n}-1/a_{n}\right)/\left(\lambda \ln2\right)\right)}^{\frac{1}{2}}-1/2a_{n}-1/2b_{n}$. The value of λ≥0 is a constant that ensures constraint (8) is met, which can be obtained cost-effectively via the bisection algorithm [32]. By obtaining the optimal transmit power variables P, they can be seen as the given input for the jamming power optimization problem in the next subsections.

### 3.2. Jamming Power Optimization

Let $c_{n}=\frac{P\left[n\right]\gamma_{0}}{d_{\mathrm{BU}}^{2}\left[n\right]}$, $d_{n}=\frac{\gamma_{0}}{d_{\mathrm{JU}}^{2}\left[n\right]}$, $e_{n}=P\left[n\right]\gamma_{0}\left|\right|w_{B}-w_{E}\left|\right|{}^{-\kappa }$ and $f_{n}=\frac{\gamma_{0}}{d_{\mathrm{JE}}^{2}\left[n\right]}$. With given P, $q_{U}$, and $q_{J}$, we can reformulate problem (13) as

(15) $$\begin{array}{ccc} \; & \max_{Q}\sum_{n=1}^{N}\left[\log_{2}\left(1+\frac{c_{n}}{1+d_{n}Q\left[n\right]}\right)-\log_{2}\left(1+\frac{e_{n}}{1+f_{n}Q\left[n\right]}\right)\right] & \end{array}$$

s.t.(9).

Problem (15) is a non-convex problem because of the non-convex objective function, which is actually difficult to solve for general *N*. However, the first term in (15) is convex w.r.t. Q[n], and thus it can be approximated to a convex function within each iteration by applying the SCA method. It is known that the first-order Taylor expansion can be used to obtain the global under-estimator for any convex function at any point [32]. Thus, denoted by $Q^{l}=\left\{Q^{l}\left[n\right],n\in N\right\}$, the given local point in the *l*-th iteration, we have

(16) $$\log_{2}\left(1+\frac{c_{n}}{1+d_{n}Q\left[n\right]}\right)\geq A_{n}^{l}+B_{n}^{l}\left(Q\left[n\right]-Q^{l}\left[n\right]\right)$$

where $A_{n}^{l}=\log_{2}\left(1+\frac{c_{n}}{1+d_{n}Q^{l}\left[n\right]}\right)$ and

$$B_{n}^{l}=\frac{-c_{n}d_{n}}{\ln2\left(1+d_{n}Q^{l}\left[n\right]\right)\left(1+c_{n}+d_{n}Q^{l}\left[n\right]\right)}.$$

With (16), problem (15) is lower bounded by the following problem for any given $Q^{l}$,

(17) $$\begin{array}{cc} \; & \max_{Q}\sum_{n=1}^{N}\left[B_{n}^{l}Q\left[n\right]-\log_{2}\left(1+\frac{e_{n}}{1+f_{n}Q\left[n\right]}\right)\right] \end{array}$$

s.t.(9).

Observe that this subproblem is concave w.r.t. Q[n] and thus can be solved efficiently by the interior-point method [32].After solving problem (17), the obtained jamming power Q serves as the given variables for the trajectory optimization problem of the UAVs.

### 3.3. Trajectory Optimization of the UAV U

Even with given P, Q, and $q_{J}$, it is still hard to achieve the optimal solution to problem (13), due to the non-concavity of the objective function w.r.t. $q_{U}$ and the non-convexity of the constraint (3). To tackle this subproblem, we first introduce the slack variables $\alpha =\{\alpha \left[n\right]={\left(H-H_{B}\right)}^{2}+||q_{U}\left[n\right]-w_{B}|{|}^{2},n\in N\}$ and $\beta =\{\beta \left[n\right]=||q_{U}\left[n\right]-q_{J}\left[n\right]|{|}^{2},n\in N\}$. After some simple transformations, solving problem (13) is equivalent to solve the following problem,

(18) $$\begin{array}{ccc} \; & \max_{q_{U},\alpha ,\beta }\sum_{n=1}^{N}\left[\log_{2}\left(1+\frac{P\left[n\right]\gamma_{0}}{\alpha [n]}+\frac{Q\left[n\right]\gamma_{0}}{\beta [n]}\right)-\log_{2}\left(1+\frac{Q\left[n\right]\gamma_{0}}{\beta [n]}\right)\right] & \end{array}$$

(19) $$\begin{array}{cc} \; & s.t.\;\alpha \left[n\right]\geq {\left(H-H_{B}\right)}^{2}+\left|\right|q_{U}\left[n\right]-w_{B}{\left|\right|}^{2}, \end{array}$$

(20) $$\begin{array}{cc} \; & \;\beta \left[n\right]\leq \left|\right|q_{U}\left[n\right]-q_{J}\left[n\right]{\left|\right|}^{2},\\ \; & \;(1)-(3). \end{array}$$

In fact, if α[n] (β[n]) is increased (decreased), the objective value of problem (13) will be decreased, and thus the constraints for α and β must satisfy the equalities. Problem (18) is still non-convex, because of the non-convex objective function in (18), and the constraints in (3) and (20). To tackle this difficulty, an important lemma is provided as below.

**Lemma** **1.** *Given $K_{1}>0$ and $K_{2}>0$, the function $f\left(x,y\right)=\log_{2}\left(1+\frac{K_{1}}{x}+\frac{K_{2}}{y}\right)$ is jointly convex w.r.t. x>0 and y>0.*

**Proof.**  See Appendix A. □

Based on Lemma 1, it is easy to prove the convexity of the first term in problem (18). By using the first-order Taylor expansions of a convex function f(x,y) in a neighborhood of $\left(x,y\right)=\left(x_{0},y_{0}\right)$, i.e., $f\left(x,y\right)=f\left(x_{0},y_{0}\right)+f_{x}\left(x_{0},y_{0}\right)\left(x-x_{0}\right)+f_{y}\left(x_{0},y_{0}\right)\left(y-y_{0}\right)$, the first term in (18) at given local points denoted by $\alpha^{l}=\left\{\alpha^{l}\left[n\right],n\in N\right\}$ and $\beta^{l}=\left\{\beta^{l}\left[n\right],n\in N\right\}$ in the *l*-th iteration, can be given as follows,

(21) $$\log_{2}\left(1+\frac{P\left[n\right]\gamma_{0}}{\alpha [n]}+\frac{Q\left[n\right]\gamma_{0}}{\beta [n]}\right)\geq \log_{2}C_{n}^{l}-\frac{D_{n}^{l}}{C_{n}^{l}\ln2}$$

where $C_{n}^{l}=1+\frac{P\left[n\right]\gamma_{0}}{\alpha^{l}\left[n\right]}+\frac{Q\left[n\right]\gamma_{0}}{\beta^{l}\left[n\right]}$ and $D_{n}^{l}=P\left[n\right]\gamma_{0}{\left(\alpha^{l}\left[n\right]\right)}^{-2}\left(\alpha \left[n\right]-\alpha^{l}\left[n\right]\right)+Q\left[n\right]\gamma_{0}{\left(\beta^{l}\left[n\right]\right)}^{-2}\left(\beta \left[n\right]-\beta^{l}\left[n\right]\right)$. Similarly, by using the first-order Taylor expansion at the given local point denoted by $q_{U}^{l}=\left\{q_{U}^{l}\left[n\right],n\in N\right\}$ in the *l*-th iteration, the convex function $\left|\right|q_{U}\left[n\right]-q_{J}\left[n\right]{\left|\right|}^{2}$, w.r.t. $q_{U}\left[n\right]$ in problem (3) and in problem (20) can be replaced by their convex lower bounds, i.e.,

(22) $$\begin{array}{ccc} \; & \left|\right|q_{U}\left[n\right]-q_{J}{\left[n\right]||}^{2}\geq \left|\right|q_{U}^{l}\left[n\right]-q_{J}\left[n\right]{\left|\right|}^{2}+2{\left(q_{U}^{l}\left[n\right]-q_{J}\left[n\right]\right)}^{T}\left(q_{U}\left[n\right]-q_{U}^{l}\left[n\right]\right). & \end{array}$$

As a result, by applying SCA technique in each iteration, we approximate the original convex functions to more manageable functions at given local points. Therefore, with (21)–(22), we have the following optimization problem

(23) $$\max_{q_{U},\alpha ,\beta }\sum_{n=1}^{N}\left[-\frac{D_{n}^{l}}{C_{n}^{l}\ln2}-\log_{2}\left(1+\frac{Q\left[n\right]\gamma_{0}}{\beta [n]}\right)\right]$$

(24) $$\begin{array}{cc} \; & s.t.\;\beta \left[n\right]\leq \left|\right|q_{U}^{l}\left[n\right]-q_{J}\left[n\right]{\left|\right|}^{2}\;+2{\left(q_{U}^{l}\left[n\right]-q_{J}\left[n\right]\right)}^{T}\left(q_{U}\left[n\right]-q_{U}^{l}\left[n\right]\right), \end{array}$$

(25) $$\begin{array}{ccc} \; & \;d_{\min}^{2}\leq \left|\right|q_{U}^{l}\left[n\right]-q_{J}\left[n\right]{\left|\right|}^{2}\;+2{\left(q_{U}^{l}\left[n\right]-q_{J}\left[n\right]\right)}^{T}\left(q_{U}\left[n\right]-q_{U}^{l}\left[n\right]\right), & \\ \; & \;\;(1),(2),(19). \end{array}$$

It is observed that problem (23) is now convex with all convex constraints. As such, the interior-point method can be used efficiently to solve this problem. Note that the lower bounds obtained by the Taylor expansions suggest that the optimal objective value by solving problem (23) is a lower bound of that of problem (18). In the next subsection, the solved $q_{U}$ is input to the trajectory optimization problem of the UAV J as the given variable.

### 3.4. Trajectory Optimization of the UAV J

With given P, Q, and $q_{U}$, we let $\delta =\{\delta \left[n\right]=H^{2}+||q_{J}\left[n\right]-w_{E}|{|}^{2},n\in N\}$, to tackle the non-concavity of the objective function w.r.t. $q_{J}$. Therefore, problem (13) can be rewritten as

(26) $$\begin{array}{cc} \; & \max_{q_{J},\beta ,\delta }\sum_{n=1}^{N}[\log_{2}\left(\beta \left[n\right]+c_{n}\beta \left[n\right]+Q\left[n\right]\gamma_{0}\right)-\log_{2}\left(\beta \left[n\right]+Q\left[n\right]\gamma_{0}\right)\\ \; & \;\;\;-\log_{2}\left(1+\frac{e_{n}\delta \left[n\right]}{\delta \left[n\right]+Q\left[n\right]\gamma_{0}}\right)] \end{array}$$

(27) $$\begin{array}{cc} \; & s.t.\;\delta \left[n\right]\geq H^{2}+||q\left[n\right]-w_{E}{\left|\right|}^{2},\\ \; & \;(1)\text{–}(3),(20). \end{array}$$

Also, the constraint for δ holds with equalities, otherwise the objective value of problem (13) will be decreased by increasing δ[n]. Similarly, by using the first-order Taylor expansion at given local points denoted by $\delta^{l}=\left\{\delta^{l}\left[n\right],n\in N\right\}$, $\beta^{l}=\left\{\beta^{l}\left[n\right],n\in N\right\}$ and $q_{J}^{l}=\left\{q_{J}^{l}\left[n\right],n\in N\right\}$ in the *l*-th iteration, the second and third terms in problem (26), and $\left|\right|q_{U}\left[n\right]-q_{J}\left[n\right]{\left|\right|}^{2}$ in (3) and in (20) can be substituted by their respective concave upper and convex lower bounds, i.e.,

(28) $$\begin{array}{cc} \log_{2}\left(\beta \left[n\right]+Q\left[n\right]\gamma_{0}\right) & \leq \log_{2}\left(\beta^{l}\left[n\right]+Q\left[n\right]\gamma_{0}\right)+\frac{\beta \left[n\right]-\beta^{l}\left[n\right]}{\ln2(\beta^{l}\left[n\right]+Q\left[n\right]\gamma_{0})}, \end{array}$$

(29) $$\log_{2}\left(1+\frac{e_{n}\delta \left[n\right]}{\delta \left[n\right]+Q\left[n\right]\gamma_{0}}\right)\leq E_{n}^{l}+F_{n}^{l}\left(\delta \left[n\right]-\delta^{l}\left[n\right]\right),$$

where $E_{n}^{l}=\log_{2}\left(1+\frac{e_{n}\delta^{l}\left[n\right]}{\delta^{l}\left[n\right]+Q\left[n\right]\gamma_{0}}\right)$,

$$F_{n}^{l}=\frac{e_{n}\gamma_{0}Q\left[n\right]}{\ln2\left(\gamma_{0}Q\left[n\right]+\left(e_{n}+1\right)\delta^{l}\left[n\right]\right)\left(\gamma_{0}Q\left[n\right]+\delta^{l}\left[n\right]\right)},$$

and

(30) $$\begin{array}{ccc} \; & \left|\right|q_{U}\left[n\right]-q_{J}{\left[n\right]||}^{2}\geq \left|\right|q_{U}\left[n\right]-q_{J}^{l}\left[n\right]{\left|\right|}^{2}-2{\left(q_{U}\left[n\right]-q_{J}^{l}\left[n\right]\right)}^{T}\left(q_{J}\left[n\right]-q_{J}^{l}\left[n\right]\right). & \end{array}$$

With problems (28)–(30), we approximate problem, (26) as the following optimization problem

(31) $$\begin{array}{ccc} \; & \max_{q_{J},\beta ,\delta }\sum_{n=1}^{N}\left[\log_{2}\left(\beta \left[n\right]+c_{n}\beta \left[n\right]+Q\left[n\right]\gamma_{0}\right)-F_{n}^{l}\delta \left[n\right]-\frac{\beta [n]}{\ln2(\beta^{l}\left[n\right]+Q\left[n\right]\gamma_{0})}\right] & \end{array}$$

(32) $$\begin{array}{cc} \; & s.t.\;d_{\min}^{2}\leq \left|\right|q_{U}\left[n\right]-q_{J}^{l}\left[n\right]{\left|\right|}^{2}\;-2{\left(q_{U}\left[n\right]-q_{J}^{l}\left[n\right]\right)}^{T}\left(q_{J}\left[n\right]-q_{J}^{l}\left[n\right]\right), \end{array}$$

(33) $$\begin{array}{cc} \; & \;\beta \left[n\right]\leq |q_{U}\left[n\right]-q_{J}^{l}\left[n\right]{\left|\right|}^{2}\;-2{\left(q_{U}\left[n\right]-q_{J}^{l}\left[n\right]\right)}^{T}\left(q_{J}\left[n\right]-q_{J}^{l}\left[n\right]\right),\\ \; & \;(1),(2),(27). \end{array}$$

Problem (31) is now a convex optimization problem that can be cost-effectively solved by the interior-point method. Furthermore, the Taylor expansions in problems (28)–(30) indicate that the objective value of problem (26) is at least the same as that by solving problem (31). Note that all the obtained variables P, Q, $q_{U}$, and $q_{J}$ are utilized as the given variables for the next iteration.

### 3.5. Overall Algorithm

In summary, the overall algorithm for obtaining the locally optimal solution to problem (13) is computed by the joint optimization of both the BS’s transmit power P and the UAV J’s jamming power Q as well as the two UAVs’ trajectories $q_{U}$, and $q_{J}$ variables, via alternatively solving subproblems (14), (17), (23) and (31) in an iterative way, respectively. The detailed procedure for solving problem (13) is summarized in Algorithm 1.

In the following, we analyze the computation complexity of Algorithm 1. In each iteration, the BS’s transmit power, UAV J’s jamming power, and the trajectories of UAVs U and J are optimized in sequence, based on the interior-point method by using existing solvers, such as CVX [33]. Therefore, the complexity for solving the four subproblems can be expressed by O(logN), $O(N^{3.5}\log\left(1/\epsilon \right))$, $O({\left(3N\right)}^{3.5}\log\left(1/\epsilon \right))$, and $O({\left(3N\right)}^{3.5}\log\left(1/\epsilon \right))$, respectively, for the given solution precision of ϵ>0 [34]. In addition, as the complexity for updating all variables in BCD iterations is in the order of log(1/ϵ), the total computation complexity of the proposed algorithm is $O(N^{3.5}\log^{2}\left(1/\epsilon \right))$. Due to the polynomial time complexity, Algorithm 1 is applicable to the aerial cooperative jamming for cellular-enabled UAV networks.

**Algorithm 1** Proposed algorithm for solving problem (13)1: Initial P, Q, $q_{U}$, $q_{J}$, α,β and δ. Let l=0. 2: **repeat** 3:  Solve problem (14) with given $Q^{l}$, $q_{U}^{l}$, and $q_{J}^{l}$,  and denote by $P^{l+1}$ the optimal solution. 4:  Solve problem (17) with given $P^{l}$, $q_{U}^{l}$, and $q_{J}^{l}$,  and denote by $Q^{l+1}$ the optimal solution. 5:  Solve problem (23) with given $P^{l}$, $Q^{l}$, $q_{J}^{l}$, $\alpha^{l}$ and $\beta^{l}$,  and denote by $q_{U}^{l+1}$ the optimal solution. 6:  Solve problem (31) with given $P^{l}$, $Q^{l}$, $q_{U}^{l}$, $\beta^{l}$ and $\delta^{l}$,  and denote by $q_{J}^{l+1}$ the optimal solution. 7:  Update **l=l+1.** 8: **until** Converge to a pre-specified precision ϵ>0.

Next, the convergence of Algorithm 1 is discussed as follows. Let $\psi (P^{l},Q^{l},q_{U}^{l},q_{J}^{l})$ denote the value of the objective function in problem (13) in the *l*-th iteration. Then, we have

(34) $$\psi \left(P^{l},Q^{l},q_{U}^{l},q_{J}^{l}\right)\leq \psi_{P}\left(P^{l+1},Q^{l},q_{U}^{l},q_{J}^{l}\right),$$

where $\psi_{P}\left(P^{l+1},Q^{l},q_{U}^{l},q_{J}^{l}\right)$ is defined as the obtained objective value of problem (14) and $P^{l+1}$ is the optimal solution to problem (14). For the optimization of the jamming power Q, the following equations hold,

(35) $$\begin{array}{cc} \psi (P^{l+1},Q^{l},q_{U}^{l},q_{J}^{l}) & \overset{\left(j_{1}\right)}{=}\psi_{Q}^{\mathrm{lb}}\left(P^{l+1},Q^{l},q_{U}^{l},q_{J}^{l}\right)\\ \; & \overset{\left(j_{2}\right)}{\leq }\psi_{Q}^{\mathrm{lb}}\left(P^{l+1},Q^{l+1},q_{U}^{l},q_{J}^{l}\right)\\ \; & \overset{\left(j_{3}\right)}{\leq }\psi \left(P^{l+1},Q^{l+1},q_{U}^{l},q_{J}^{l}\right), \end{array}$$

where $\psi_{Q}^{\mathrm{lb}}$ is denoted as the objective value of problem (17), ($j_{1}$) holds since the first-order Taylor expansion in (16) is tight at the local point $Q^{l}$ in problem (17), ($j_{2}$) satisfies due to the optimal solution $Q^{l+1}$ to problem (17), and ($j_{3}$) is because the computed objective value of problem (17) is lower bounded by that of problem (15). For the two UAVs’ trajectories optimization, the similar derivation procedure as in (35) can be used, which are given as below,

(36) $$\begin{array}{cc} \psi (P^{l+1},Q^{l+1},q_{U}^{l},q_{J}^{l}) & =\psi_{q_{U}}^{\mathrm{lb}}\left(P^{l+1},Q^{l+1},q_{U}^{l},q_{J}^{l}\right)\\ \; & \leq \psi_{q_{U}}^{\mathrm{lb}}\left(P^{l+1},Q^{l+1},q_{U}^{l+1},q_{J}^{l}\right)\\ \; & \leq \psi (P^{l+1},Q^{l+1},q_{U}^{l+1},q_{J}^{l}), \end{array}$$

(37) $$\begin{array}{cc} \psi (P^{l+1},Q^{l+1},q_{U}^{l+1},q_{J}^{l}) & =\psi_{q_{J}}^{\mathrm{lb}}\left(P^{l+1},Q^{l+1},q_{U}^{l+1},q_{J}^{l}\right)\\ \; & \leq \psi_{q_{J}}^{\mathrm{lb}}\left(P^{l+1},Q^{l+1},q_{U}^{l+1},q_{J}^{l+1}\right)\\ \; & \leq \psi (P^{l+1},Q^{l+1},q_{U}^{l+1},q_{J}^{l+1}), \end{array}$$

With (34)–(37), we finally obtain that

(38) $$\psi \left(P^{l},Q^{l},q_{U}^{l},q_{J}^{l}\right)\leq \psi \left(P^{l+1},Q^{l+1},q_{U}^{l+1},q_{J}^{l+1}\right).$$

As a result, Algorithm 1 ensures that the obtained objective value of problem (13) is non-decreasing over the iterations, and thus it guarantees its convergence to the locally optimal solution to problem (13).

## 4. Numerical Results

In this section, we verify our joint trajectories and powers optimization (denoted as 2T&P) algorithm through simulations. Three benchmark schemes are taken into account as a comparison:

- UAVs’ trajectories optimization without power control (denoted as 2T/NP);
- heuristic UAVs’ trajectories with power control (2HT/P);
- joint optimization of the UAV U’s trajectory and BS’s power control without aerial cooperative jamming from the UAV J (denoted as 1T&P), which is identical with the algorithm proposed in [18].

Specifically, the 2T/NP scheme sets the powers of the BS and the UAV U as $P\left[n\right]=\bar{P}$ and $Q\left[n\right]=\bar{Q},\forall n$, respectively, and the trajectories of the two UAVs are obtained by solving problems (23) and (31) iteratively until convergence. In the 2HT/P scheme, the UAV U flies directly to the top of the BS at its maximum speed, then stays hovering as long as possible, and finally travels directly to its destination at its maximum speed by the end of *T*. Different from UAV U, UAV J keeps hovering right above the eavesdropper with the largest achievable rate. Given heuristic trajectories in the 2HT/P, the powers P[n] and Q[n] can be obtained by solving problems (14) and (17), respectively. The initial UAV trajectory for the 2T&P and 2T/NP schemes are constructed by the heuristic UAV trajectories as in 2HT/P. The simulation parameters are specified in Table 1.

**Figure 2:**
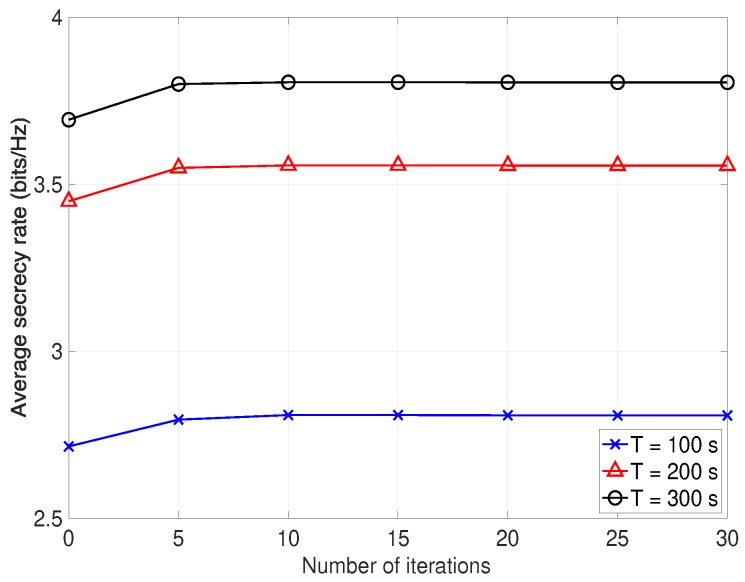
The convergence performance of the proposed Algorithm 1.

We first verify the convergence behaviour of the proposed Algorithm 1 versus the iteration numbers for different *T* in Figure 2. It is illustrated that the average secrecy rate increases quickly and converges within five iterations, and its performance increases significantly with *T*. This confirms that a locally optimal solution to problem (13) can be converged by using the proposed algorithm.

**Figure 3:**
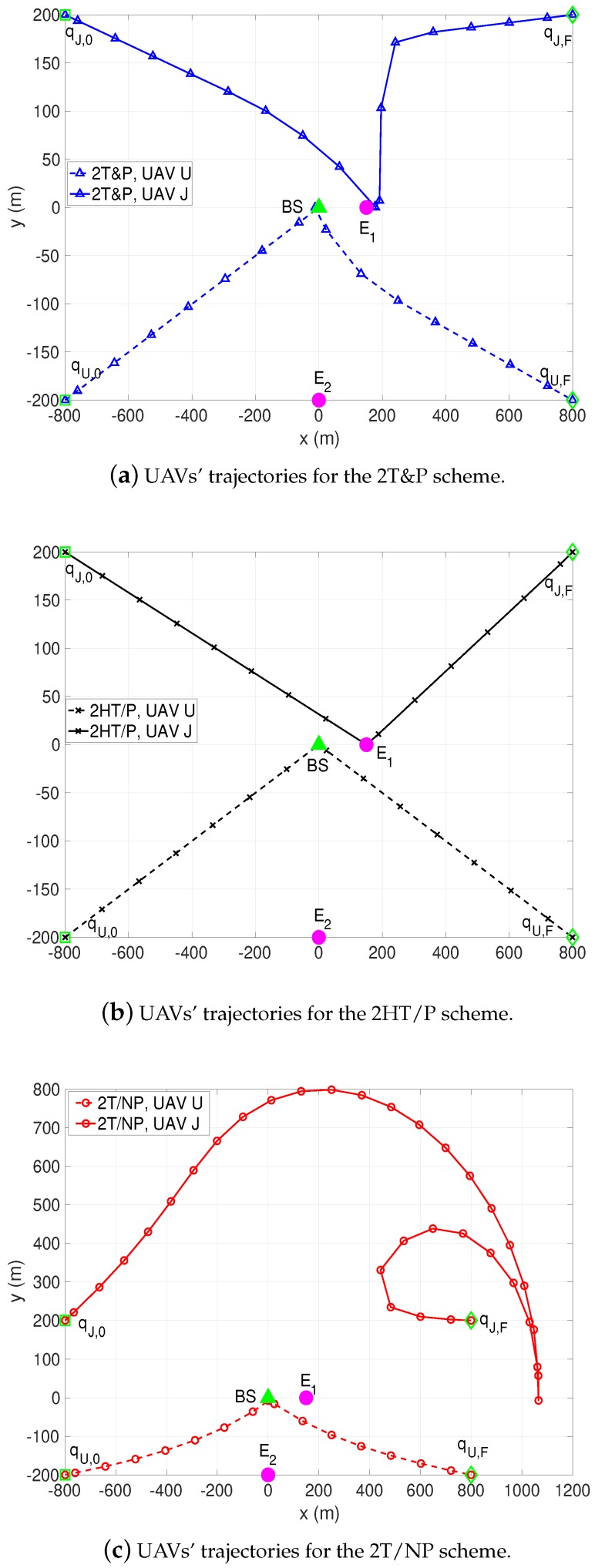
UAVs’ trajectories by different schemes for T=300 s. All trajectories are sampled every 5 s. The horizontal locations of the BS, eavesdroppers, UAVs’ initial and final locations are marked with ▲, ⚫, ☐ and ◇, respectively.

Figure 3 illustrates the optimized trajectories of the two UAVs by different schemes when *T* is sufficiently large, e.g., T=300 s. It is observed that the hovering locations of all algorithms for the UAV U are directly above the BS. This occurs because the locations of the eavesdroppers are not related to the UAV U’s trajectory due to the ground-to-air transmission, and thus the UAV U can obtain its maximum achievable rate hovering at the location on top of the BS. In addition, the the trajectories of the UAV U in 2T&P and 2T/NP show the curved paths in order to escape from the unintended interference caused by the UAV J. However, the trajectories of the UAV J present significant different. In particular, for our 2T&P scheme in Figure 3a, the UAV first flies along an arc-like path and reaches a certain point close to the eavesdropper $E_{1}$ to avoid a collision with the UAV U; then, it keeps static at this hovering location for a permission period, and finally reaches its destination by the end of *T*, also in an arc-like path to prevent it causing much interference for the UAV U. Notice that the hovering location of the UAV J is closer to $E_{1}$ compared to $E_{2}$, as the channel quality of BS-to-$E_{1}$ link is much better than that of BS-to-$E_{2}$ link. The BS-to-$E_{2}$ link can also be degraded if the UAV J can guarantee that the secrecy of the worst-case, i.e., BS-to-$E_{1}$ link transmission, by taking advantage of the dominant air-to-ground links. Moreover, at their hovering locations, the UAVs can achieve the better secrecy rate by effectively balancing between enhancing the communication of the ground-to-air link and degrading the quality of the BS-to-$E_{i}$ channel. In contrast with the 2T&P scheme, we can observe that on its way to the final location, the UAV J flies in a big arc path to keep away from the UAV U in the 2T/NP scheme as shown in Figure 3c. This is because the BS’ s transmit power and UAV J’s jamming power in 2T/NP are fixed, and thus the UAV J has to fly as far as possible to avoid severe interference with the UAV U over the whole duration, *T*.

Note that there is a tradeoff between improving the average achievable secrecy rate of the UAV U and avoiding the interference induced by the UAV J. For 2HT/P in Figure 3b, with the pre-specified UAV trajectories, the BS and the UAV jammer can adjust their power allocations to enhance the secrecy performance. Specifically, the BS gradually increases its transmit power before the UAV U flies to its hovering location, while the UAV J properly decreases its jamming power when it reaches above $E_{1}$ to suppress the interference to the UAV U. In contrast, a secure communication-aware UAV trajectory design provides additional flexibility to avoid interference between UAVs in our 2T&P scheme. Thus, the UAV J adaptively adjusts its jamming power and trajectory according to the BS’s transmit power and the location of the UAV U to further achieve the better secrecy rate.

**Figure 4:**
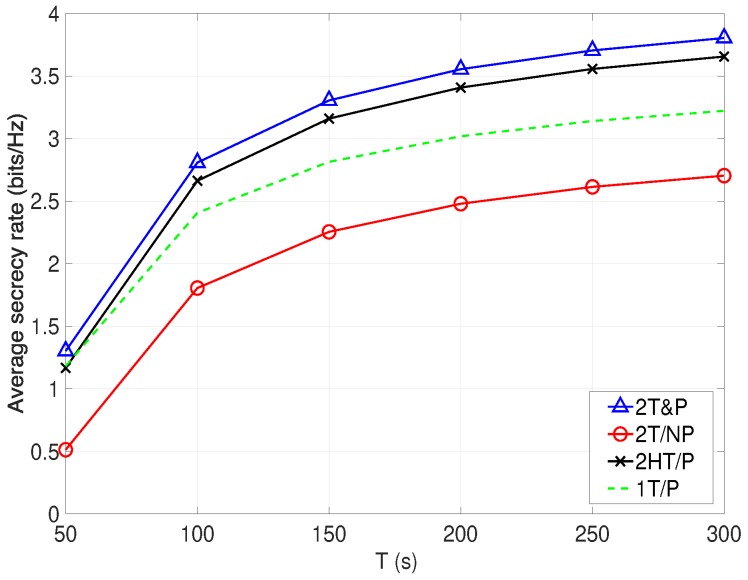
Average secrecy rate versus *T* with different trajectory and power control designs.

Figure 4 illustrates the average secrecy rate versus *T*. It is expected that the average secrecy rates obtained by all schemes raise with *T*, and the proposed 2T&P scheme significantly outperforms other benchmark schemes owing to its joint optimization. Moreover, the proposed 2T&P scheme provides the significant gain as compared to the scheme in [18], i.e., 1T&P. This indicates that the advantage brought by the aerial cooperative jamming is more effective and important on notably improving the average secrecy rate. However, the 2T/NP presents the worst performance, which demonstrates that the power control also plays a key role in avoiding jamming from other UAVs, which is necessary in our cellular-enabled UAV communication networks with aerial cooperative jamming; otherwise, the secrecy rate can be significantly degraded as shown in Figure 4. Note that we expect that the proposed 2T&P algorithm can still achieve the best secrecy performance via the joint design, even if the number of the eavesdropper increases. This is because the joint design guarantees that the eavesdropper with the best channel condition can be effectively jammed by the UAV J; other eavesdroppers cannot wiretap confidential messages from the BS. The obtained results validate the advantages of introducing aerial cooperative jamming, and the joint optimization of UAV trajectories and power allocations.

## 5. Conclusions

Integrating UAVs into the forthcoming 5G cellular networks faces new security challenges. Thus, a new type of cooperative aerial jamming scheme for the cellular-enabled UAV secure communication networks has been investigated in this paper. In particular, the UAV receiver and the UAV jammer cooperate closely with each other to maximize the worst-case average secrecy rate by jointly optimizing their trajectories and the BS/UAV transmit/jamming power. An efficient iterative solution has been proposed to approximately tackle the secrecy rate maximization problem over a given flight period, by means of the BCD and SCA methods. The proposed algorithm is guaranteed to converge to a locally optimal solution with suitable computational complexity. We have demonstrated, by numerical results, that the friendly UAV jammer provides flexible mobility for interference with the ground eavesdroppers, as well as effective power control of preventing it from jamming the UAV receiver, and thereby improves the system secrecy performance. Furthermore, the proposed scheme significantly outperforms the benchmark schemes with simple heuristic trajectories and pre-configured powers. The current scenario can also be extended to the general case with multiple legitimate UAVs, where optimal communication scheduling between the BS and each UAV should be considered. In this case, the design for UAV trajectories needs to avoid collision between UAVs more effectively, and reconciles a tradeoff between maximizing the minimum secrecy rate among multiple UAVs and suppressing the interference from the UAV jammer, which is an interesting problem to be resolved in the future.

## Figures and Tables

**Table 1:** Simulation parameters.

Notation	Physical Meaning	Simulation Value
$q_{U,0}$	Initial horizontal location of the UAV U	${\left[-800,-200\right]}^{T}$ m
$q_{U,F}$	Final horizontal location of the UAV U	${\left[800,-200\right]}^{T}$ m
$q_{J,0}$	Initial horizontal location of the the UAV J	${\left[-800,200\right]}^{T}$m
$q_{J,F}$	Final horizontal location of the UAV J	${\left[800,200\right]}^{T}$ m
$w_{B}$	Horizontal location of the BS	${\left[0,0\right]}^{T}$ m
$w_{E_{1}}$	Location of the first eavesdropper	${\left[150,0\right]}^{T}$ m,
$w_{E_{2}}$	Location of the second eavesdropper	${\left[-200,0\right]}^{T}$ m
*H*	Altitude of UAVs	100 m
$H_{B}$	Altitude of BS	10 m
$V_{\max}$	Maximum speed of UAVs	40 m/s
$d_{\min}$	Minimum safe distance between UAVs	20 m
$\delta_{t}$	Time slot length	1 s
$\rho_{0}$	Channel power gain at the reference distance	−60 dB
$\sigma^{2}$	Noise power levels	−110 dBm
$\bar{P}$ and $\hat{P}$	Average and peak power of UAV U	20 dBm and 26 dBm
$\bar{Q}$ and $\hat{Q}$	Average and peak power of UAV J	10 dBm and 16 dBm
ϵ	Accuracy threshold	$10^{-4}$

## Appendix A. Appendix

We prove Lemma 1 via the definition of convex functions. First, since $f\left(x,y\right)=\log_{2}\left(1+\frac{K_{1}}{x}+\frac{K_{2}}{y}\right)$ where x>0, y>0, $K_{1}>0$ and $K_{2}>0$, the first-order derivatives of f(x,y) w.r.t. *x* and *y* are given by

(A1) $$f_{x}\left(x,y\right)=-\frac{K_{1}}{x^{2}G\ln2},\;f_{y}\left(x,y\right)=-\frac{K_{2}}{y^{2}G\ln2},$$

where we let $G=1+\frac{K_{1}}{x}+\frac{K_{2}}{y}$ for brevity. Then, the Hessian of f(x,y) is

(A2) $$\nabla^{2}f\left(x,y\right)=\left[\begin{array}{cc} \frac{2K_{1}xy+2K_{1}K_{2}x+K_{1}^{2}y}{x^{4}yG^{2}\ln2} & -\frac{K_{1}K_{2}}{x^{2}y^{2}G^{2}\ln2}\\ -\frac{K_{1}K_{2}}{x^{2}y^{2}G^{2}\ln2} & \frac{2K_{2}xy+2K_{1}K_{2}y+K_{2}^{2}x}{xy^{4}G^{2}\ln2} \end{array}\right]$$

For any $t={\left[t_{1},t_{2}\right]}^{T}$, we have

$$t^{T}\nabla^{2}f\left(x,y\right)t=\frac{F_{1}\left(x,y\right)+F_{2}\left(x,y\right)+F_{3}\left(x,y\right)}{x^{4}y^{4}G^{2}\ln2}\geq 0$$

where $F_{1}\left(x,y\right)=2K_{1}t_{1}^{2}xy^{3}\left(y+K_{2}\right)$, $F_{2}\left(x,y\right)=2K_{2}t_{2}^{2}x^{3}y\left(x+K_{1}\right)$ and $F_{3}\left(x,y\right)={\left(t_{1}K_{1}y^{2}-t_{2}K_{2}x^{2}\right)}^{2}$ for x>0, y>0, $K_{1}>0$ and $K_{2}>0$, which finally leads to the convexity of the function f(x,y).
